# Transfer of 4-hydroxynonenal from parasitized to non-parasitized erythrocytes in rosettes. Proposed role in severe malaria anemia

**DOI:** 10.1111/j.1365-2141.2011.09015.x

**Published:** 2012-04

**Authors:** Sophie Uyoga, Oleksii A Skorokhod, Michael Opiyo, Emily N Orori, Thomas N Williams, Paolo Arese, Evelin Schwarzer

**Affiliations:** 1 Department of Genetics, Biology and Biochemistry, University of Torino Medical SchoolTorino, Italy; 2 Kenya Medical Research Institute/Wellcome Trust Research ProgrammeKilifi, Kenya; 3 Department of Paediatrics, Oxford Radcliffe NHS Trust, University of OxfordOxford, UK

**Keywords:** malaria, malarial anaemia, rosette, 4-hydroxynonenal

## Abstract

Severe anaemia is a life-threatening complication of *falciparum* malaria associated with loss of predominantly non-parasitized red blood cells (npRBCs). This poorly elucidated process might be influenced by (i) rosettes, i.e. npRBCs cytoadherent to haemozoin-containing parasitized RBCs (pRBCs) and (ii) generation in pRBCs of 4-hydroxynonenal (4-HNE) through haemozoin-catalysed lipid peroxidation. We explored whether close proximity in rosettes may facilitate 4-HNE transfer to npRBCs, which is likely to enhance their phagocytosis and contribute to malaria anaemia. Fluorescence microscopy and flow cytometry data indicated 4-HNE transfer to npRBCs in rosettes. Rosettes were formed by 64·8 ± 1·8% varO-expressing pRBCs, and 8·7 ± 1·1% npRBCs were positive for 4-HNE-protein-conjugates, while low-rosetting parasites generated only 2·4 ± 1·1% 4-HNE-conjugate-positive npRBCs. 4-HNE transfer decreased after blocking rosetting by monoclonal antibodies. A positive linear relationship between rosette frequency and 4-HNE-conjugates in npRBCs was found in 40 malaria patients, a first indication for a role of rosetting in npRBCs modifications *in vivo.* Children with severe malaria anaemia had significantly higher percentages of 4-HNE-conjugate-positive npRBCs compared to children with uncomplicated malaria. In conclusion, 4-HNE transfer from pRBCs to npRBCs in rosettes is suggested to play a role in the phagocytic removal of large numbers of npRBCs, the hallmark of severe malaria anaemia.

Rosetting is a specialized form of cytoadherence of late haemozoin (HZ)-containing forms of *Plasmodium falciparum*-parasitized red blood cells (pRBCs) to non-parasitized (np)RBCs. Rosetting intensity is positively associated with malaria complications, such as severe malaria anaemia and cerebral malaria (Rowe *et al*, 1995; Mercereau-Puijalon *et al*, 2008; Mayor *et al*, 2011). While microvascular obstruction, tissue hypoxia and acidosis resulting from rosetting and cytoadherence to endothelia may contribute to cerebral malaria (Dondorp *et al*, 2004), the mechanisms by which rosetting provokes severe malaria anaemia, notably in the hallmark massive removal of npRBCs, is unexplored. Membrane lipid modifications, including a decreased content of polyunsaturated fatty acids (PUFAs) arachidonic and docosahexaenoic acid, have been described in np-RBCs co-cultured with p-RBCs (Beaumelle & Vial, 1988; Omodeo-Sale *et al*, 2003). Natural HZ (nHZ), produced in large amounts in mature pRBCs and conservatively isolated from pRBC cultures, has a crystalline scaffold of ferriprotoporphyrin IX molecules from undigested haemoglobin haem bound to host and parasite proteins and lipids, notably PUFAs (Schwarzer *et al*, 1996, 2003). Haem iron of HZ is able to peroxidize PUFAs and generate the terminal aldehyde 4-hydroxynonenal (4-HNE) present in micromolar concentrations in late parasite forms and able to diffuse to adjacent cells (Skorokhod *et al*, 2007, 2010). In previous studies, supplemented low-micromolar 4-HNE dose-dependently formed covalent conjugates with a number of proteins (adducts and Schiff bases) and decreased cell deformability of normal RBCs (Skorokhod *et al*, 2007).

In this study we explored whether 4-HNE may diffuse from pRBC to npRBCs in rosettes, providing a mechanistic explanation for the induced damage to and the enhanced removal of npRBCs and for the association between rosetting and severe malaria anaemia (Rowe *et al*, 1995; Mercereau-Puijalon *et al*, 2008; Doumbo *et al*, 2009). We also performed *ex vivo* studies in patients presenting with clinical malaria to see whether the frequency of rosetting parasites correlated with the percentage of npRBCs carrying 4-HNE conjugates. Finally, we examined whether the frequency of npRBCs carrying 4-HNE conjugates was higher in anaemic than in non-anaemic children.

## Materials and methods

All chemicals were from Sigma (Sigma-Aldrich, St. Louis, MO, USA) if not otherwise stated.

### In vitro culture of varO 89F5 P. falciparum in human RBCs

The varO expressing variant of the 89F5 *P. falciparum* strain (varO parasites, a well-characterized rosetting parasite line, provided by O. Mercereau-Puijalon, Pasteur Institute, Paris, France) was utilized in this study. The varO parasites were maintained under 5% O_2_, 5% CO_2_ and 90% N_2_ atmosphere in group O Rh^+^ RBCs at 1% haematocrit in growth medium (GM; RPMI 1640 medium supplemented with 20 mmol/l HEPES, 2 mmol/l glutamine, 10% (vol/vol) AB^+^ serum, 0·025 mmol/l adenine, 20 mmol/l glucose, 32 μg/ml gentamicin). Cultures were maintained at a rosette frequency of at least 50% by weekly enrichment by centrifugation (30 s at 660 ***g***) of trophozoite-stage cultures through ice-cold ficoll (1·077 g/ml; Seromed, Berlin, Germany). Cultures were followed up for rosette frequency and varO expression as described below. To obtain parasite cultures with different rosetting frequencies of the same parasite line, after separation on ficoll, varO cultures were divided into high and low rosetting subpopulations, which were found in pellet and supernatant respectively, and kept in separate cultures.

### Analysis of 4-HNE transfer in rosettes

To determine the role of rosettes for 4-HNE transfer from p- to npRBCs, in a first approach schizonts from high and low rosetting cultures were enriched to >90% parasitaemia at the 40/60% interface of a discontinuous percoll gradient as described (Schwarzer *et al*, 2003). Parasites were collected and re-infection started by adding washed RBCs from freshly drawn blood from healthy donors (blood group O Rh^+^) adjusting parasitaemia to 2%. The cultures were kept under rotation at 40 rpm. At 40-h post-reinfection, rosette frequency, varO expression in mature parasites, parasitaemia and 4-HNE conjugates on the npRBCs were assayed. As the 4-HNE-protein conjugates in npRBC were not significantly increased (19·5 ± 1·8%; *N* = 3, *P* = 0·25) with increasing parasitaemia (15 vs. 5%) in *in vitro* cultures, all *in vitro* assays were performed at defined parasitaemias between 2·5 and 5% to exclude this rosette-independent variation. Parasitaemias did not differ between low and high rosetting cultures after one re-infection cycle.

In a second approach, the ability of varO cultures to rosette was blocked by the addition of the blocking mouse monoclonal antibody (mAb) against the rNTS-DBL1α domain of varO (varO-MAB) (Vigan-Womas *et al*, 2008) (gift from O. Mercereau-Puijalon, Pasteur Institute) at 150 ng/ml 12 h post-reinfection and the 4-HNE transfer to npRBCs was analysed and compared to non-blocked rosetting varO cultures at 40 h.

Additionally, the poorly permeant HNE–trap *N*-acetylcysteine (NAC) was used to show that 4-HNE bound to npRBCs most likely originated from the 4-HNE-rich pRBCs. To this end, npRBCs were co-cultured with trophozoite-parasitized RBCs 30 h after reinfection in the presence or absence of 1 mmol/l NAC. Cultures were kept at 2·5–5% parasitaemia and 1% haematocrit for 12 h. Thereafter co-cultured npRBCs were analysed for 4-HNE conjugates.

### Assay of rosetting rate

Rosette frequency (the proportion of trophozoites that were found in rosettes, counting at least 200 trophozoites) was determined as described (Rowe *et al*, 1997) by fluorescence microscopy [Leica DR IRB fluorescence microscope equipped with a Leica DFC 420C camera, a 63× oil planar apochromatic objective with 1·32 numerical aperture, a 515/560 nm excitation and LP 590 nm barrier filter and the version 3.3.1 of the Leica DFC image software (Leica Microsystems, Wetzlar, Germany)] in ethidium bromide-stained (5 μg/ml) wet smears.

### Assay of varO expression

pRBCs were assayed for expression of the varO-antigen which mediates the binding of trophozoites to npRBCs in the rosette. For this, aliquots were washed with phosphate-buffered saline (PBS) containing 5 mmol/l glucose and 2% bovine serum albumin (PBSGlc-BSA), subsequently incubated with varO-MAB at a concentration of 50 μg/ml (gift from O. Mercereau-Puijalon, Pasteur Institute) in PBSGlc-BSA for 1 h (Vigan-Womas *et al*, 2008) washed with PBSGlc-BSA, and incubated with secondary fluorescein isothiocyanate (FITC)–conjugated goat anti mouse IgG at a dilution of 1:400 and 5 μg/ml ethidium bromide (for pRBC labelling) for 1 h. The washed, stained cells were analysed for FITC in the FL1 channel at 515–545 nm and for ethidium bromide in the FL2 channel at 564–606 nm after argon laser excitation at 488 nm using a FACSCalibur flow cytometer (BD Biosciences, Sunnyvale, CA, USA) and FlowJo™ 9.0.2. software (Tree Star Inc., Ashland, OR, USA).

### Assay of 4-HNE conjugates

Surface expression of 4-HNE conjugates on npRBCs was determined by flow cytometry and fluorescence microscopy. For flow cytometry, cells were washed with PBSGlc-BSA, incubated with rabbit anti-4-HNE serum (Alexis Biochemicals, Lausen, Switzerland) at 1:50 in PBSGlc-BSA for 1 h, washed twice with PBSGlc-BSA, and incubated with secondary FITC-conjugated goat anti-rabbit FITC-conjugated antibody (1:400) as previously indicated (Skorokhod *et al*, 2010) and 5 μg/ml ethidium bromide (for labelling pRBCs) for 1 h. The washed labelled cells were analysed for FITC in the FL1 channel at 515–545 nm and for ethidium bromide in the FL2 channel at 564–606 nm after argon laser excitation at 488 nm using FACSCalibur flow cytometer (BD Biosciences) and the softwares FlowJo™ 9.0.2. software (Tree Star Inc.) and WinMDI (The Scripps Research Institute, La Jolla, CA, USA). Ethidium bromide-negative cells were gated and analysed for FITC labelling. Washed, freshly collected RBCs of healthy donors were incubated in parallel in the absence of pRBC and treated similar to the controls to set the cut-off for HNE-positivity.

Additional analysis of 4-HNE conjugates was performed by fluorescence microscopy (Leica Microsystems) in wet smears following incubation with primary rabbit anti 4-HNE serum (Alexis) and secondary anti-rabbit IgG*-*ATTO488 antibody produced in goat at a dilution of 1:200. Fluorescence images were acquired with a fluorescence microscope [Leica DR IRB fluorescence microscope equipped with a Leica DFC 420C camera, a 63× oil planar apochromatic objective with 1·32 numerical aperture, a 450/490 nm excitation and LP 515 nm barrier filter and the version 3.3.1 of the Leica DFC image software (Leica Microsystems)]. Trophozoites were identified by the presence of HZ in bright field inspection and confirmed by ethidium bromide staining.

### In vitro erythrophagocytosis assay

Erythrophagocytosis was quantified by flow cytometry after feeding stained RBCs to stimulated phagocytes of the human monocytic cell line THP-1. RBCs, incubated or not with 4-HNE, were washed in PBSGlc, adjusted to 15% haematocrit and stained with 0·4 mg/ml eosine-5-maleimide in the dark at room temperature for 1 h. The RBCs were then spun, the supernatant containing the unbound dye carefully removed and RBCs washed three times with PBSGlc-BSA. Opsonization of RBCs with IgG anti-D (Rhophylac, ZLB Behring, King of Prussia, PA, USA) at 750 units/ml packed RBCs was performed at 30% haematocrit at 37°C for 30 min with subsequent washes in PBSGlc-BSA. THP-1 cells were grown in suspension in RPMI 1640 medium, supplemented with 10% heat-inactivated fetal bovine serum (FBS), 100 units/ml penicillin and 100 μg/ml streptomycin (complete medium). Before starting phagocytosis, the THP-1 cells were stimulated with γ-interferon (IFN-γ, 50 units/ml) and tumour necrosis factor α (TNF-α, 250 units/ml) for 24 h to enhance their phagocytic activity. Then, cells were washed three times with RPMI 1640 medium and suspended in complete medium. Phagocytosis was started by adding a) stained or non-stained, b) opsonized or non-opsonized RBCs at a RBC/phagocyte ratio of 200:1, and performed at 37°C in a humidified CO_2_/air-incubator for 3 h. Thereafter, the cell suspension was loaded on Ficoll to separate THP-1 cells from non-ingested RBCs by centrifugation at 900 ***g*** for 20 min. THP-1 cells were harvested from the top of the Ficoll and washed with complete medium and their fluorescence was measured by FACSCalibur flow cytometer in the FL2 channel at 564–606 nm after excitation at 488 nm and analysed with CellQuest (BD Biosciences) or WinMDI (Scripps Research Institute) software. THP-1 cells acquired fluorescence with phagocytosed RBCs at discrete intensities corresponding to discrete numbers of phagocytosed RBCs. Mean fluorescence intensity of stained RBCs was used as the reference for quantifying phagocytosis by THP-1 cells.

### Ex vivo assay of rosettes and 4-HNE conjugates in natural P. falciparum infections

Blood was collected following individual informed consent from children aged 1–12 years who were admitted with *P. falciparum* malaria to Kilifi District Hospital, Kenya, between July and September 2010. Standard haematological parameters were assessed by routine methods. Severe malaria anaemia was defined by haemoglobin values ≤50 g/l and a parasite-positive blood smear. Peripheral blood mononuclear cells, platelets and neutrophils were removed from freshly drawn whole blood and pRBCs matured in culture for 24 h before being assayed for rosette and 4-HNE conjugate frequencies as described above. Ethical permission for this study was received from the KEMRI/National Ethical Review Committee in Nairobi.

### Statistical analysis

The analysis of variance (anova) test was performed to compare data obtained with the *in vitro* parasite cultures (Microcal Origin 5.0; Microcal Software, Northampton, MA, USA). Independent *t*-tests were performed to compare means between independent groups and determine significance of differences. *P*-values of 0·05, 0·01 and 0·0037 were used to define statistical significance and are given where relevant. Linear regression analysis was performed to determine the linear dependency of two parameters. Prism software, version 5.0, from GraphPad Inc. (San Diego, CA, USA) was used.

## Results

### 4-HNE transfer in rosettes in varO cultures in vitro

**Figure 1:**
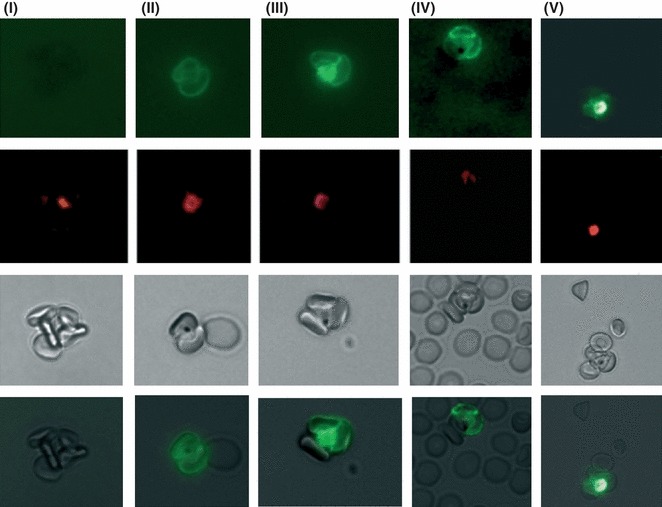
4-HNE transfer from parasitized to non-parasitized RBCs in rosettes *in vitro*. Micrographs of 4-HNE-positive parasitized RBCs (pRBCs) and non-parasitized RBCs (npRBCs) in rosettes from high varO expressing *P. falciparum* cultures. RBCs from synchronized parasite cultures at trophozoite stage and 2% parasitaemia were stained either with rabbit anti-4-HNE-conjugate antibody and ATTO488-conjugated anti-rabbit secondary antibody to reveal 4-HNE-conjugates with RBC membrane proteins (upper lane, panels ii–v) or with secondary antibody alone to define non-specific background (upper lane, panel i). Fluorescence images from rosettes were acquired with a Leica DR IRB fluorescence microscope equipped with a 63× oil planar apochromatic objective (panels i–v). Panels in the second lane show the same rosettes stained with ethidium bromide, panels in the third lane show bright field images with the central HZ-containing trophozoite. Panels in the bottom lane show merged images of lanes one and three.

Sub-populations of the varO parasite culture, characterized by varying varO expression and rosette frequencies, were analysed in order to determine the role of rosettes in 4-HNE transfer from pRBCs- to npRBCs. We have previously shown that trophozoites generate 4-HNE conjugates on their surface due to HZ-elicited lipoperoxidation (Skorokhod *et al*, 2007). An intense labelling for 4-HNE conjugates, evidenced by fluorescence microscopy of *in vitro* cultured varO trophozoites, was confirmed in the current study. Typically, the fluorescent, occasionally very bright trophozoite within the rosette was surrounded by npRBCs displaying distinct fluorescence (Fig 1ii–v). The trophozoite was unequivocally identified by the HZ crystals and ethidium bromide staining (Fig 1, lane 2, 3 and merged images in lane 4). Within the rosettes, <5% npRBCs had no detectable 4-HNE conjugates (see Fig 1iii, where one of three rosetting npRBCs was not labelled). In contrast, npRBCs that were not entrapped in rosettes were mostly unlabelled for 4-HNE conjugates (Fig 1iv, v).

**Figure 2:**
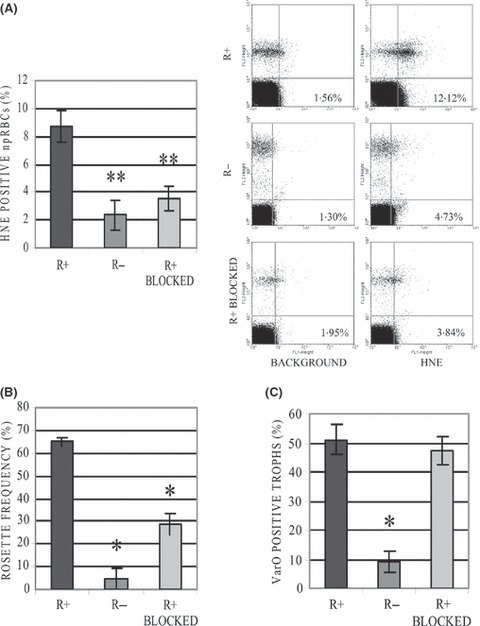
4-HNE conjugate formation in non-parasitized RBCs (npRBCs) co-cultured *in vitro* with trophozoites depends on rosette frequency. High (R+) and low rosetting (R−) cultures and cultures in which varO was blocked by anti-varO-MAB (R+ BLOCKED) were analysed for 4-HNE conjugate positivity in npRBCs (A), rosetting frequency (B) and varO expression (C) at trophozoite stage. The percentage of ethidium bromide–negative npRBCs that stained positive for 4-HNE conjugates was quantified by flow cytometry. Representative dot plots of the 4-HNE conjugate analysis are shown. Rosette frequency was determined by fluorescence microscopy as the percentage of trophozoites found in rosettes of ethidium bromide-labelled cultures. VarO expression on trophozoites was analysed with mouse monoclonal antibody against the rNTS-DBL1α domain of varO by flow cytometry. Columns represent mean percentages, bars are standard errors of the means and the number of samples assayed are 7, 3 and 3 for R+, R− and R+BLOCKED, respectively. The statistically significant differences between R+ and R− or R+ BLOCKED are indicated (*P < 0·01; **P < 0·05).

The role of rosettes in the transfer of 4-HNE from pRBCs to npRBCs was verified by flow-cytometry analysis of 4-HNE conjugates on npRBCs from cultures of the same parasite strain with high and low varO expression and rosette frequency, respectively. In high varO expressing cultures with 51·1 ± 5·3% (mean ± SE, *N* = 7) varO-positive trophozoites, 64·9 ± 1·8% (*N* = 6) of trophozoites formed rosettes and 8·7 ± 1·2% (*N* = 10) of co-cultured npRBCs were positive for 4-HNE conjugates. By contrast, in low-rosetting cultures with only 9·1 ± 3·5% (*N* = 3) of varO-positive trophozoites and a rosetting rate of 4·7 ± 4·6% (*N* = 3), the portion of 4-HNE-positive npRBCs was significantly lower and only 2·4 ± 1·1% (*N* = 3, *P* = 0·017) despite similar trophozoite parasitaemias (Fig 2A–C). The strong decrease in 4-HNE transfer to npRBCs seen in low *versus* high varO cultures, was recapitulated when the rosetting frequency of varO-positive trophozoites was decreased to 28·9 ± 4·5% (*N* = 3) using blocking mAbs raised against the rNTS-DBL1α domain of the varO antigen. The percentage of 4-HNE-positive npRBCs decreased significantly to 3·5 ± 0·8% (*N* = 3) under this condition (Fig 2A), suggesting that the intimate cell–cell contact within the rosettes may result in a more intense 4-HNE transfer between cells compared to the occasional short term contacts between non-rosetting cells in the rotating cell culture system.

Additional evidence that 4-HNE was indeed transferred from p- to np-RBCs was provided by the remarkable inhibition of 4-HNE-conjugation in npRBC by the 4-HNE trap *N*-acetylcysteine (NAC), added to co-cultured p- and np-RBCs. In the presence of NAC, rosetting pRBCs were no longer able to significantly increase the share of 4-HNE-positive npRBCs, while in absence of NAC, a fourfold increase was observed compared to control npRBCs (*n* = 3, *P* = 0·04).

### 4-HNE induced RBC phagocytosis in vitro

**Figure 3:**
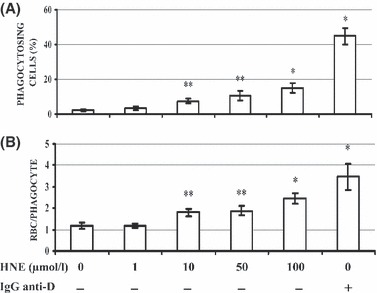
Increased phagocytosis of npRBCs after 4-HNE supplementation. RBCs were incubated with 4-HNE at indicated final concentrations for 30 min and washed thereafter. RBCs were stained with eosine-5-maleimide at 15% haematocrit in the dark at room temperature for 1 h, washed and fed to THP-1 phagocytes for 3 h. For positive phagocytosis control, RBCs were opsonized with IgG anti-D and stained with eosine-5-maleimide as detailed in Materials and methods. For negative phagocytosis control, eosine-5-maleimide-stained RBCs without further treatments were fed to phagocytes. The share of THP1 cells that have taken up one or more 4-HNE- or IgG anti-D-treated RBCs or untreated RBCs (panel A) as well as the numbers of RBCs taken up by a single phagocyte (panel B) were determined by flow cytometry. Columns are means and bars standard errors. Independent phagocytosis experiments were performed with 4-HNE-treated RBCs from three donors and with untreated- and IgG anti-D-treated RBCs from four donors. The significances of differences to untreated control RBCs are indicated (*P < 0·01; **P < 0·05).

The addition of 0–100 μmol/l 4-HNE to npRBC dose–dependently increased the share of phagocytosing THP-1 cells as well as the number of RBCs taken up by a single phagocyte (Fig 3). Significant differences were observed from 10 μmol/l upwards. At 100 μmol/l 4-HNE, npRBCs were phagocytosed by 15 ± 2·9% (mean ± SE, *n* = 3) THP-1 cells at 2·5 ± 0·4 RBCs per cell (mean ± SE, *n* = 3). Phagocytosis-positive cells reached one-third of that observed when anti-D IgG opsonized RBCs were phagocytosed, the latter being a widely used control for high phagocytosis.

### In malaria patients, the frequency of rosetting parasites correlates with the percentage of 4-HNE-modified npRBCs

**Figure 4:**
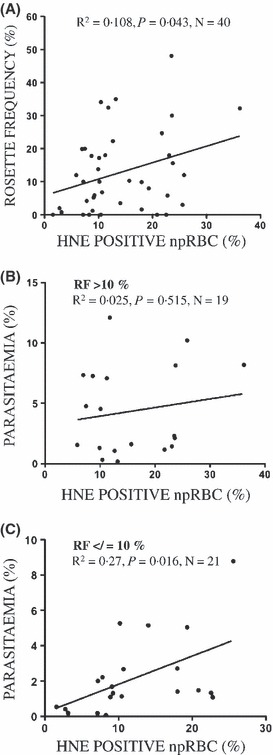
4-HNE transfer from parasitized to non-parasitized RBCs in rosettes from patients. (A) Correlation between rosette frequency and percentage of 4-HNE-positive non-parasitized RBCs (npRBCs) in patients with acute malaria infection. (B) No correlation between parasitaemia and percentage of 4-HNE-positive npRBCs in patients with acute infection with rosetting parasite phenotypes. (C) Correlation between parasitaemia and percentage of 4-HNE-positive npRBCs in patients with acute infection with non/low rosetting parasite phenotypes. RBCs from patients with acute malaria infection were kept in culture for 24 h to obtain trophozoite stages, before rosetting frequencies and percentages of 4-HNE-positive npRBCs were measured. Rosette frequency was determined by fluorescence microscopy as the percentage of trophozoites found in rosettes of ethidium bromide-labelled cultures. The percentage of ethidium bromide-negative npRBCs that were positive for 4-HNE conjugates was quantified by flow cytometry. The parameter pairs of 40 patients were plotted in (A) 19 of them with rosetting frequencies (RF) > 10% in (B) and 21 with RF ≤ 10% in (C) and the regression line shows the association between both parameters.

**Figure 5:**
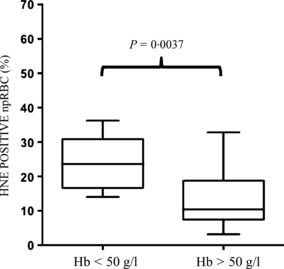
Association of 4-HNE conjugate formation with anaemia. The percentage of ethidium bromide-negative non-parasitized RBCs (npRBCs) that were positive for 4-HNE conjugates was quantified in two groups of patients with haemoglobin concentrations either below 50 g/l (Hb < 50 g/l, *N* = 5) or above 50 g/l (Hb > 50 g/l, *N* = 33) and plotted in the respective group. The box plots show the median (central line), the 25th and 75th percentiles (box) and minimum and maximum values (whiskers). 4-HNE conjugate formation was significantly different between both groups, *P* < 0·0037.

The significance of 4-HNE transfer in rosettes seen in the *in vitro* cultures was investigated in malaria patients with 0·2–12% parasitaemia. A positive linear relationship (*R*^2^ = 0·108, *P* = 0·043, *N* = 40) was found between rosette frequency and 4-HNE conjugates in npRBCs (Fig 4A), indicating that infections by the rosetting phenotype may significantly increase the membrane modifications by 4-HNE in npRBCs *in vivo*, leading to the increased removal of these modified cells. In case of infection with high-rosetting phenotypes and rosetting rates above 10% in the blood, the 4-HNE conjugate formation was parasitaemia-independent (Fig 4B). At low rosette frequencies only, parasitaemia determined the 4-HNE-conjugate formation (Fig 4C). Finally, a significantly higher percentage of npRBCs was positive for 4-HNE among children presenting with severe anaemia (defined as a haemoglobin concentration of <50 g/l) (23·75 ± 3·68%, *N* = 5) than in non-anaemic children (12·85 ± 1·25%, *N* = 33, *P* = 0·0037) (Fig 5).

## Discussion

pRBCs cytoadhere to a number of host tissues and circulating blood cells, notably to npRBCs (Rowe *et al*, 2009). Cytoadhesion of pRBCs to npRBCs is defined as ‘rosetting’ (Udomsangpetch *et al*, 1989; Wahlgren *et al*, 1992). Rosettes are dynamic structures of varying dimensions, frequently with <10 npRBCs per 1-2 pRBCs and rarely with up to more than 50 npRBCs (Wahlgren *et al*, 1992; Mercereau-Puijalon *et al*, 2008). The major parasite ligand in cytoadherence and rosetting is the variant *P. falciparum* erythrocyte membrane protein 1 (PfEMP1), expressed on the surface of pRBCs (Mercereau-Puijalon *et al*, 2008; Rowe *et al*, 2009). PfEMP1 has a large extracellular domain with a sequence of six adhesion modules called Duffy-Binding Like (DBL) domains DBLα, β, γ, δ, ε and X (Mercereau-Puijalon *et al*, 2008; Rowe *et al*, 2009). In the rosette-forming laboratory strain Palo Alto varO used in this study, rosetting is mediated by the N-terminal DBL1α domain of PfEMP1 (Mercereau-Puijalon *et al*, 2008; Juillerat *et al*, 2010). The major receptors for the varO strain on the npRBC surface are blood group antigens, glycosaminoglycans and heparansulphate-like molecules (Mercereau-Puijalon *et al*, 2008).

Rosettes are stable cell aggregates temporarily disrupted in arterial capillaries but immediately re-formed in venous capillaries and larger vessels, where they withstand circulatory shear stress (Kaul *et al*, 1991; Wahlgren *et al*, 1992). Rosetting has been associated with all clinical forms of severe malaria in a number of studies (Rowe *et al*, 1995; Mercereau-Puijalon *et al*, 2008; Doumbo *et al*, 2009; Mayor *et al*, 2011).

While significantly impaired microvascular blood flow with metabolic disturbances due to rosettes (Kaul *et al*, 1991) may explain the pathogenesis of organ malaria, such as cerebral malaria, it is evidently inadequate to explain the massive removal of npRBCs, a hallmark of severe malaria anaemia, which results from three elements: lysis of pRBCs at schizogony, impaired erythropoiesis and removal of npRBCs (Casals-Pascual & Roberts, 2006). The latter exceeds the lysis of pRBCs by approximately 10-fold and is thus considered to be the most important factor leading to anaemia (Jakeman *et al*, 1999).

The present data, obtained *in vitro* and validated by *ex vivo* studies in patients, may provide a mechanism that explains how pRBCs damage adjacent npRBCs in rosettes and target those to phagocytic removal. Such a mechanism is based on the transfer of 4-HNE from pRBCs to npRBCs in rosettes. 4-HNE, produced in pRBCs as a final product of PUFAs peroxidation mediated by nHZ (Schwarzer *et al*, 2003; Skorokhod *et al*, 2007), has been shown to diffuse out of the producing cells either into the blood or extracellular fluid, or to adjacent cytoadherent cells where it may rapidly form covalent conjugates with cysteine, lysine and histidine residues in proteins (for review see Poli *et al*, 2008). For example, 4-HNE was shown to diffuse from nHZ-laden monocytes to adjacent erythroid precursors and to inhibit their maturation to RBCs (Skorokhod *et al*, 2010). Binding of 4-HNE to proteins is very rapid, reaching a maximum approximately 3 min after 4-HNE supplementation to isolated cells (Siems & Grune, 2003). Due to its lipophilic tail, 4-HNE preferentially concentrates in the membrane of the target cells. In fact, we have shown that when npRBCs are supplemented with low-micromolar concentrations of 4-HNE, conjugates were formed with membrane or cytoskeletal proteins, such as spectrin, band 3, and actin (Table SI) and resulted in decreased RBC deformability (Skorokhod *et al*, 2007) and enhanced RBC phagocytosis (Fig 2). Thus, 4-HNE production in pRBCs may account for 4-HNE binding to membrane proteins of npRBCs in rosettes followed by increased membrane rigidity and their flagging as damaged cells and phagocytic removal by organ phagocytes, contributing to the development of malarial anaemia.

In varO-positive cultures with a rosette frequency of 64·9%, 8·7% of co-cultured npRBCs were 4-HNE-positive, while only 2·4% of co-cultured npRBCs were 4-HNE-positive in the low-rosetting culture. Additionally, blocking the rosetting capacity of trophozoites by antibodies resulted in decreasing the rosette frequency by about 56% and of 4-HNE-labelling of npRBCs by about 60%. The fourfold greater positivity for 4-HNE conjugates in the npRBCs of the high-rosetting culture may explain a significantly higher phagocytic removal of those npRBCs.

Data obtained from patients admitted with acute malaria infection are in agreement with the above sequence and mechanism. First, *ex vivo* data obtained with separated RBCs from these patients kept in culture for 24 h to obtain mature rosetting parasites, demonstrate a significant positive correlation between the percentage of 4-HNE-positive npRBCs and rosette frequency. In patients with the high-rosetting phenotype, 4-HNE-membrane modifications in npRBCs were independent of parasitaemia. In fact, the vast majority (75%) of those patients displayed 4-HNE conjugates on more than 10% of their np-RBCs. In contrast, non- or low-rosetting parasites elicited similar rates of 4-HNE conjugates only in 50% of patients. This strengthens the idea that the rosette formation consistently contributes to the oxidative modification of npRBCs *in vivo*, and indicates that direct transfer of 4-HNE produced in the maturing parasite is facilitated by the intimate contact of p- and npRBCs in the rosette. As to the remarkable formation of 4-HNE conjugates *in vivo*, additional factors, such as cytoadherence of p- and npRBCs during extensive microvascular obstructions observed in severe malaria *in vivo*, may contribute to 4-HNE formation and transfer of 4-HNE conjugates in npRBCs (Schmidt *et al*, 1996; Dondorp *et al*, 2008). Second, the percentage of npRBCs positive for 4-HNE conjugates was significantly higher in patients presenting with anaemia than in their non-anaemic counterparts, confirming that oxidative membrane modifications – probably driven by rosette formation- may play a role in the excess removal of npRBCs in malaria anaemia.

In conclusion, we have shown that 4-HNE, a HZ-generated terminal product of PUFA peroxidation, can be transferred from pRBCs to npRBCs in rosettes. As normal RBCs treated with plausible amounts of 4-HNE were altered and intensely phagocytosed, it is proposed that rosettes may be instrumental in eliciting the removal of large numbers of npRBCs, a characteristic hallmark of severe malaria anaemia. Malaria patients’ data are in agreement with this mechanism.

## Supporting Information

**Table SI.** Formation of HNE-conjugates with cytoskeleton proteins after supplementation of npRBC with 10 lmol/l HNE.
