# Supplementary material for: Transfer of 4-hydroxynonenal from parasitized to non-parasitized erythrocytes in rosettes. Proposed role in severe malaria anemia
Source: Br J Haematol. 2012 Apr;157(1):116–24. doi: 10.1111/j.1365-2141.2011.09015.x (PMC3412292; doi:10.1111/j.1365-2141.2011.09015.x)
Supplement: Supplementary file 1 [file bjh0157-0116-SD1.pdf]

Table S1. Formation of HNE-conjugates with cytoskeleton proteins after supplementation of npRBC with 10µM HNE.

*Protein	ID Swiss-Prot/ Uniprot	number of identified Michael adducts	number of identified Schiff bases
Spectrin alpha (280 014 Da)	P02549	19	8
Spectrin beta (246 468 Da)	P11277	51	18
Band 3 anion transport protein (101 792 Da)	P02730	6	1
Actin, cytoplasmic 1 (ACTB, 41 737 Da)	P60709	Work in progress	Work in progress

*Proteins were in-gel digested by trypsin overnight at 37 °C. The peptides were extracted from the gel pieces, mixed with an equal volume of 10 mg/ml α-cyano-4-hydroxy-cinnamic acid in 40% (v/v) acetonitrile/0.1% (v/v) trifluoroacetic acid and spottet as a microcrystalline thin film onto a 96-spot MALDI target. MALDI-mass spectrometry was performed using a Micromass TofSpec 2E spectrometer (Manchester, UK), equipped with a 337 nm nitrogen laser. The instrument operated in the positive ion reflectron mode at 20 kV accelerating voltage with time-lag focusing. Each mass spectrum was generated by accumulating data from 100 to 120 laser shots. Spectra were calibrated by close spot internal calibration using trypsin, and the proteins were identified by peptide mass fingerprinting with the free search program MASCOT 2.3.02 (http://www.matrixscience.com). The following parameters were used in the searches: taxa *Homo sapiens*, trypsin digest, one missed cleavage by trypsin, carbamidomethylation of cysteine as fixed modification, and methionine oxidation and C, H, K modification by 4-HNE as variable modifications. Maximum error allowed was 100 ppm. Identification of protein spots was performed by triplicate analysis.
